# Supplementary material for: Single-Cell Transcriptomics Reveals the Complexity of the Tumor Microenvironment of Treatment-Naive Osteosarcoma
Source: Front Oncol. 2021 Jul 21;11:709210. doi: 10.3389/fonc.2021.709210 (PMC8335545; doi:10.3389/fonc.2021.709210)
Supplement: Supplementary Document 1 — Kaplan-Meier curve of the 518 genes associated with survival. [file DataSheet_1.zip › Supplementary Document 1/sur.GPRC5C.pdf]

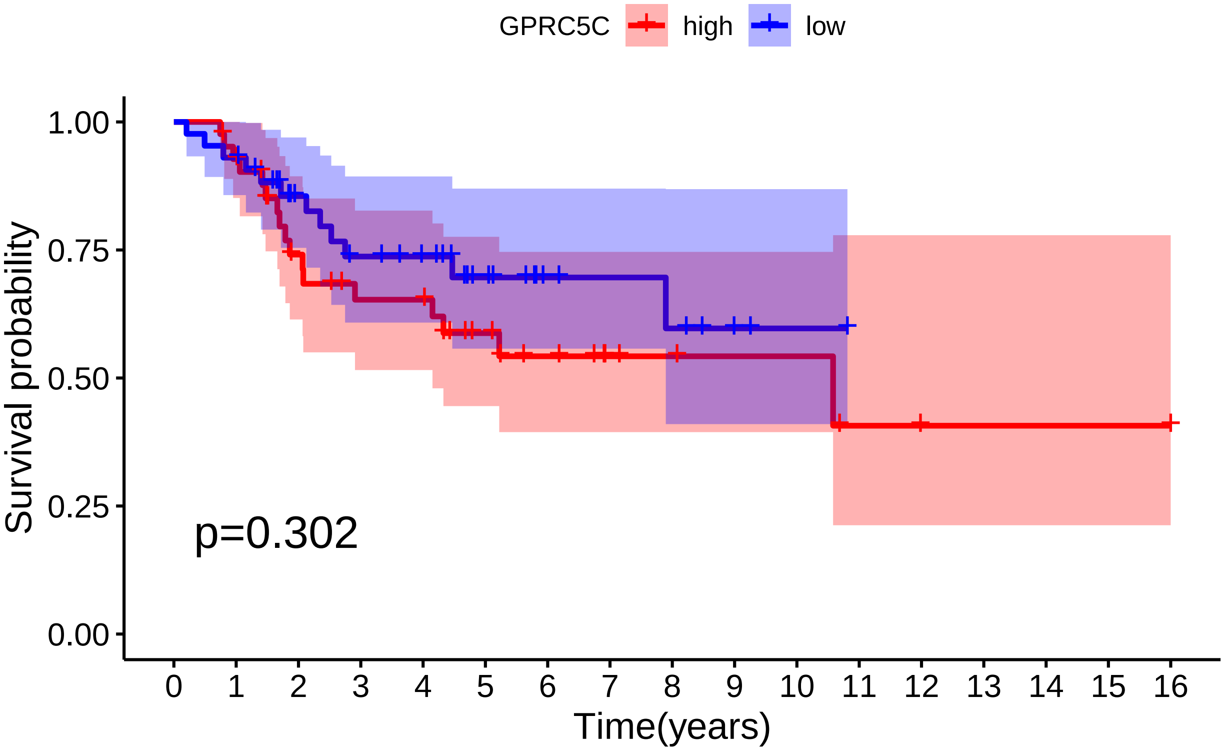

GPRC5C	0	1	2	3	4	5	6	7	8	9	10	11	12	13	14	15	16
high	42	38	26	21	21	14	10	6	5	4	4	2	1	1	1	1	1
low	43	40	29	24	21	14	8	7	6	3	1	0	0	0	0	0	0

Time(years)
